# Prescribing Justice: A Close Examination of Physicians Acting As Expert Witnesses in Saudi Arabia

**DOI:** 10.7759/cureus.56909

**Published:** 2024-03-25

**Authors:** Ghala B Albishri, Saad B Albishri, Bashaer A AlMansour, Mamdouh K Zaki

**Affiliations:** 1 Miscellaneous, College of Law, King Faisal University, Hofuf, SAU; 2 Miscellaneous, College of Medicine, Imam Mohammad Ibn Saud Islamic University, Riyadh, SAU; 3 Department of Public Law, College of Law, King Faisal University, Hofuf, SAU; 4 Forensic Medicine, Forensic Medicine Services Center, Ministry of Health, Taif, SAU

**Keywords:** medical law, saudi arabia, testimony, medical expert witness, expert witness

## Abstract

Introduction: An expert witness is a person who provides testimony on issues that the court finds to be outside the scope of their expertise and experience. Any physician who has performed an independent medical evaluation or written medical records can and should expect to be requested as an expert witness. Medical malpractice, workers' compensation, and personal injury are the most prominent areas where medical expert witnesses participate and provide opinions and testimony. To our knowledge, this is the first study to be conducted in Saudi Arabia on physicians acting as expert witnesses.

Methods: This observational descriptive cross-sectional study conducted in Saudi Arabia from November 2022 to July 2023 aimed to assess physicians' experiences, education, training, willingness, self-competency, attitude, and perception as expert witnesses. The study population consisted of physicians working in Saudi Arabia, with at least a specialist level of professional expertise. Participants completed a self-administered online survey, utilizing a researcher-designed questionnaire.

Results: In total, 417 participants, with males comprising 51.3% of the sample, responded to the survey. More than half of the physicians (58.3%) had never produced a medical report for the court. Among those who had, the majority had done so one to twice. Similarly, the majority had never testified in court (77.5%), with only a small percentage having done so once or twice. Approximately 80% of participants had no prior education or training as expert witnesses, but among those who did, courses and workshops were the most common forms of education or training. Most participants expressed interest in learning or training for this role (69.1%) and were willing to provide medical reports or court testimony (73.9%). However, half of the participants did not feel competent in writing a medical report for the court, and more than half lacked confidence in giving testimony.

Conclusion: The findings highlight the need for increased engagement, education, and training among physicians, particularly early and mid-career professionals, to enhance their confidence and competence as expert witnesses and ensure ethical practices in the medicolegal domain in Saudi Arabia.

## Introduction

An expert witness is a person who provides testimony on issues that the court finds to be outside the scope of their expertise and experience. Any physician who has performed an independent medical evaluation or written medical records can and should expect to be requested as an expert witness. Medical malpractice, workers' compensation, and personal injury are the most prominent areas where medical expert witnesses participate and provide opinions and testimony. The purpose of an expert witness is to assist the court in understanding the facts in dispute to facilitate the decision-making process. An expert witness is expected to be objective and independent and to base their judgment on the most recent advances in medical knowledge. Typically, testimony is restricted within the expert witness's expertise [1].

Worldwide studies have examined physicians' experiences and competence as expert witnesses in various specialties [2-6]. The Royal College of Psychiatrists' Continuing Professional Development Committee surveyed the current competencies and training of psychiatrists in this role. The survey received responses from 38 career-grade and 22 trainee psychiatrists. Results showed that nearly half of career-grade psychiatrists had no courtroom experience, but they had prepared psychiatric reports for the courts. Most respondents lacked formal training as expert witnesses. Around 72.4% of career-grade psychiatrists felt insufficiently trained during their residency. Among trainee psychiatrists, about half had written psychiatric court reports under supervision, and 23.8% had observed court sessions. However, most trained psychiatrists had no formal training as expert witnesses [7]. Emergency physicians' expertise as expert witnesses was assessed at an adult tertiary referral hospital in Australia. The study found that 71% had testified in court, and 65% had provided written expert opinions. A significant majority of 90% believed that specialized training in emergencies should include medical evidence training, preferably through mock court sessions and forensic workshops. Furthermore, 23% felt confident in their ability to handle courtroom trials. Overall, the study concluded that emergency physicians lacked sufficient training as expert witnesses, considering it one of their most challenging tasks, emphasizing the need for further training in this area [8]. To the best of our knowledge, no study has been conducted involving all specialties in the medical field.

In Saudi Arabia, confessions are most important as evidential submissions, requiring repetition before the judge. Testimonies from ordinary witnesses rank second, followed by expert witness testimonies [9]. Article 128 of the Ministry of Justice's law of civil procedure states that the court can assign one or more experts, specifying their tasks, report submission deadlines, hearing dates, and payment terms. Experts may provide oral opinions during hearings, which will be recorded [10]. The Code of Ethics for Healthcare Practitioners emphasizes the significance of medical witnesses in helping the judiciary understand patients' conditions and treatments, promoting honesty and objectivity in delivering medical facts [11]. A national study involving 750 healthcare practitioners in Saudi Arabia revealed that 97% had poor awareness of medical law, and nearly half had not read The Code of Ethics for Healthcare Practitioners [12]. To our knowledge, no study has been conducted in Saudi Arabia on physicians acting as expert witnesses.

This study has four aims. The first is to describe physicians' current experiences, education, and training in acting as expert witnesses in Saudi Arabia. The second is to demonstrate the willingness of physicians to serve as expert witnesses in Saudi Arabia. The third is to describe the self-competence of physicians in becoming expert witnesses in Saudi Arabia. The fourth is to demonstrate the attitude and perception of physicians toward serving as expert witnesses in Saudi Arabia.

## Materials and methods

Study design

This cross-sectional study was conducted in Saudi Arabia from November 2022 to June 2023. The study population involved physicians who are at least specialists at a professional level and working in Saudi Arabia. Forensic medicine professionals were excluded from the study since serving as an expert witness is part of their job description [9]. Participants completed a self-administered online survey. The target sample size was at least 384 based on 5% precision with a 95% confidence interval (CI). Data collectors were recruited from various regions of Saudi Arabia, including the Central, Western, Eastern, Southern, and Northern regions. The data was gathered by delivering the questionnaire directly to physicians through hospital visits. Furthermore, the survey was widely disseminated via social media. Weekly online meetings were organized to track the progress of the data collectors.

Data collection tool

There were four sections to the questionnaire. The first section included the participants' data and information about their work experience. The second section contained information about their experience as expert witnesses. The third section detailed their education and training as medical expert witnesses. The fourth section assessed their willingness, self-competency, attitude, and perceptions toward becoming medical expert witnesses. The study utilized a researcher-designed questionnaire to describe physicians' experiences, education, training, willingness, self-competency, attitude, and perception as expert witnesses. In total, the questionnaire contained 20 items.

Content and face validity

Two experts, a legal consultant, and a forensic medicine consultant, reviewed the survey questions to assess content validity. They provided feedback on comprehensiveness, readability, relevance, and appropriateness. The legal consultant, with expertise in law and courtroom procedures, critiqued and modified the questions to ensure they accurately reflected the legal aspects of expert witness testimony. They checked for appropriate legal terminology and adherence to the legal system in Saudi Arabia. The forensic medicine consultant, who wrote medicolegal reports and provided courtroom testimony as a physician, served as a vital link between the medical and legal fields. They critiqued the survey questions from a forensic standpoint, verifying their alignment with the challenges and requirements faced by physicians acting as expert witnesses in the legal system. Face validity was established by distributing the survey questionnaire to physicians from various specialties. The physicians were asked to assess the questionnaire's clarity and appropriateness based on their professional experiences in the medical field. The feedback collected from the physicians was utilized to refine and improve the survey instrument.

Statistical analysis

IBM SPSS v. 28 (IBM Corp., Armonk, NY) was used to conduct descriptive statistical analysis as the data was summarized using numbers and percentages. The correlations between categorically measured variables were evaluated using the chi-squared test of independence.

## Results

In total, 417 participants responded to the survey, with males making up 51.3% of the sample. The distribution across regions was appropriate, with the majority residing in the Central (29.5%) and Eastern (29.0%) regions. Most physicians had worked in Ministry of Health hospitals (26.6%), followed by private hospitals (25.9%). Around 54.2% of participants held consultant positions in their medical professions. Approximately one-third of respondents had five to nine years of experience in their respective specialties. The study sample predominantly comprised physicians in internal medicine, emergency medicine, family medicine, general surgery, OB/GYN, and pediatrics, accounting for 11.3%, 9.8%, 9.8%, 9.4%, 8.9%, and 8.2%, respectively. A detailed presentation of the demographic characteristics of the participants is seen in Table 1.

**Table 1:** Descriptive analysis of the participants sociodemographic characteristics. (N = 417)

Variable		N	%
Sex	Male	214	51.3%
	Female	203	48.7%
Nationality	Saudi	404	96.9%
	Non-Saudi	13	3.1%
Residence	Central Region	123	29.5%
	Eastern Region	121	29.0%
	Northern Region	42	10.0%
	Southern Region	44	10.6%
	Western Region	87	20.9%
Hospital type	Ministry of Health Hospital	111	26.6%
	Private Hospital	108	25.9%
	University Hospital	95	22.8%
	National Guard Hospital	41	9.8%
	Security Forces Hospital	32	7.7%
	Armed Forces Hospital	30	7.2%
Professional level	Consultant	226	54.2%
	Fellow	34	8.2%
	Specialist	157	37.6%
Years of practice	<5 years	93	22.3%
	5 - 9 years	141	33.8%
	10 - 14 years	64	15.3%
	15 - 20 years	60	14.4%
	>20 years	59	14.2%
Specialty	Anesthesiology	13	3.1%
	Dermatology	21	5.0%
	Emergency medicine	41	9.8%
	ENT	14	3.4%
	Family medicine	41	9.8%
	General surgery	39	9.4%
	Internal medicine	47	11.3%
	Neurosurgery	15	3.6%
	OB/GYN	37	8.9%
	Ophthalmology	21	5.0%
	Orthopedics	20	4.8%
	Pediatrics	34	8.2%
	Psychiatry	23	5.5%
	Radiology	20	4.8%
	Urology	21	5.0%
	Other	10	2.4%

Over half of the physicians (58.3%) had never produced a medical report for the court. Among those who had, 24.9% had done so one to two times. The majority had never testified in court (77.5%), with only 14.1% having done so once or twice. Approximately 80% of participants had no prior education or training as expert witnesses. Among those who did, courses (58.3%) and workshops (46.4%) were the most common forms of education or training. Table 2 demonstrates physicians’ experiences and previous education/training as medical expert witnesses. Table 3 outlines the interest, willingness, and self-competence of the study sample regarding being an expert witness. Most participants were interested in learning or training to become expert witnesses (69.1%), and 73.9% were willing to provide medical reports or court testimony. Half of the participants did not feel competent in writing a medical report for the court, and more than half lacked confidence in giving testimony.

**Table 2:** Descriptive analysis of the participants’ experience and previous education/training as a medical expert witness. (N = 417)

Variable		N	%
Written a medical report for court.	0	243	58.3%
	1-2	104	24.9%
	3 - 5	31	7.4%
	6 - 8	12	2.9%
	≥9	27	6.5%
Given testimony as a medical expert witness.	0	323	77.5%
	1-2	59	14.1%
	3 - 5	24	5.8%
	6 - 8	3	0.7%
	≥9	8	1.9%
Previous education/training for being a medical expert witness.	None	333	79.9%
	Yes	84	20.1%
	Courses	49	58.3%
	Workshop	39	46.4%
	Diploma	26	31.0%
	Journals	16	19.0%
	Lectures	1	1.2%
	Self-education	1	1.2%
	A member in a medical practice committee.	2	2.4%

**Table 3:** Descriptive analysis of the participants' interest, willingness, and self-competence as medical expert witnesses. (N = 417)

Variable		* N*	* %*
Interest in learning/training to become a medical expert witness.	Yes	288	69.1%
	No	129	30.9%
Willingness to provide a medical report/testimony for court.	Yes	308	73.9%
	No	109	26.1%
Self-competence in writing a medical report for court.	Yes	208	49.9%
	No	209	50.1%
Self-competence in giving a testimony in court.	Yes	170	40.8%
	No	247	59.2%

Table 4 illustrates the bivariate analysis of participants' interest, willingness, and self-competence based on sex, professional level, and years of practice. Female respondents showed a slightly higher willingness (78.3%) to provide medical reports or testimony in court, but their interest in learning and training as medical expert witnesses was not statistically significant (72.4%). In contrast, a significantly higher number of males felt competent in writing medical reports (56.5%) and giving court testimony (61.9%). Males had a significantly higher likelihood of feeling competent to testify in court than females (p <0.001).

**Table 4:** Descriptive bivariate analysis of the participants' interest, willingness, and self-competence with their sex, professional level, and years of practice.

Variables		n	Interest in learning/training to become a medical expert witness.			Willingness to provide a medical report/testimony for court			Feeling competent in writing a medical report for court			Feeling competent in giving a testimony in court		
			Yes; n(%)	No; n(%)	P-value	Yes; n(%)	No; n(%)	P-value	Yes; n(%)	No; n(%)	P-value	Yes; n(%)	No; n(%)	P-value
Sex	Male	214	141(65.8)	73(34.2)	P = 0.15	149(69.6)	65(30.4)	P = 0.04	121(56.5)	93(43.5)	P = 0.005	153(61.9)	94(38.1)	P < 0.001
	Female	203	147(72.4)	56(27.6)		159(78.3)	44(21.7)		87(43.0)	116(57.0)		50(29.4)	120(70.6)	
Professional level	Consultant	226	180(79.6)	46(20.4)	P < 0.001	194(85.8)	32(14.2)	P < 0.001	122(53.9)	104(46.1)	P = 0.008	86(37.9)	140(62.1)	P = 0.434
	Fellow	34	20(58.8)	14(41.2)		25(73.5)	9(26.5)		22(78.6)	12(21.4)		16(47.1)	18(52.9)	
	Specialist	157	69(43.9)	88(56.1)		89(56.7)	68(43.3)		64(40.7)	93(59.3)		68(43.2)	89(56.8)	
Years of practice	<5 years	93	59(63.4)	34(36.6)	P < 0.001	55(59.2)	38(40.8)	P < 0.001	40(43.0)	53(57.0)	P = 0.006	40(43.0)	53(57.0)	P = 0.237
	5-9 years	141	79(56.0)	62(44.0)		96(68.1)	45(31.9)		67(47.5)	74(52.5)		65(46.0)	76(54.0)	
	10-14 years	64	50(78.1)	14(21.9)		56(87.5)	8(12.5)		40(62.5)	24(37.5)		19(29.6)	45(70.4)	
	15-20 years	60	51(85.0)	9(15.0)		52(86.7)	8(13.3)		23(38.2)	37(61.8)		22(36.7)	38(63.3)	
	>20 years	59	49(83.1)	10(16.9)		49(83.1)	10(16.9)		38(64.4)	21(35.6)		24(40.6)	35(59.4)	

The professional level of participants significantly correlated with their interest, willingness, and competence in being a medical expert witness. Most consultants expressed interest in acquiring expert witness training (79.6%) and were willing to provide medical reports in court (85.8%). In contrast, a higher percentage of fellows and specialists showed disinterest in training (41.2% and 56.1%, respectively) and providing medical reports/testimonies (26.5% and 43.3%, respectively). Among consultants, 53.9% felt competent in writing medical reports, while around 37.9% felt competent in giving court testimony. Fellows followed a similar trend, with more participants feeling competent in preparing medical reports but a slightly lower number feeling competent in giving testimony. On the other hand, a significantly higher number of specialist participants felt incompetent in writing medical reports and giving testimony (59.3% and 56.8%, respectively).

Years of practice among medical professionals also showed a statistically significant correlation with interest in education/training, willingness, and feeling of competence in writing medical reports as expert witnesses. Participants with five to nine years of experience displayed the highest interest in training (56.0%) and willingness to provide medical reports/testimony (68.1%). Those with less than five years of experience also showed significant interest in education/training and providing reports/testimony (63.4% and 59.2%, respectively). Physicians with 10-14 years of experience showed less interest in acquiring education/training compared to others. Among physicians with five to nine years of experience, the highest percentage felt competent in writing medical reports for the court (47.5%) and giving court testimony (46.0%). Participants with less than five years of experience had the second-highest percentage of physicians who felt competent in preparing medical reports and giving court testimony.

**Figure 1:**
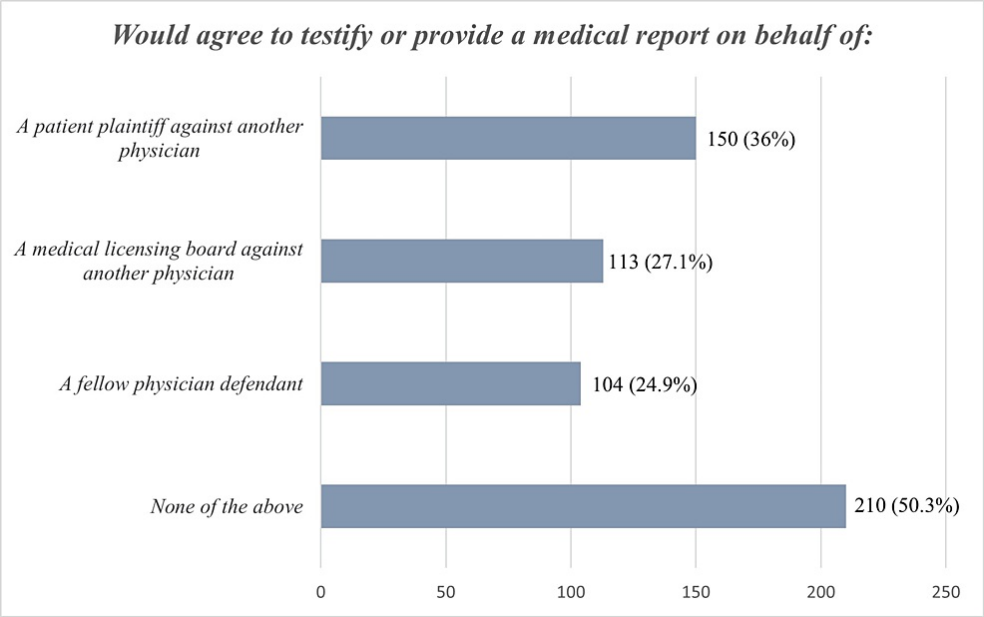
Attitude of physicians towards representing certain plaintiffs/defendants.

Figure 1 depicts the attitude of physicians towards representing certain plaintiffs/defendants. The bulk of study participants would not accept providing testimony or a medical report on behalf of a patient plaintiff against another physician, on behalf of a medical licensing board against another physician, or on behalf of a fellow physician defendant. Nevertheless, out of the three instances, there was the most consensus on testifying or providing a medical report on behalf of a patient plaintiff against another physician.

**Figure 2:**
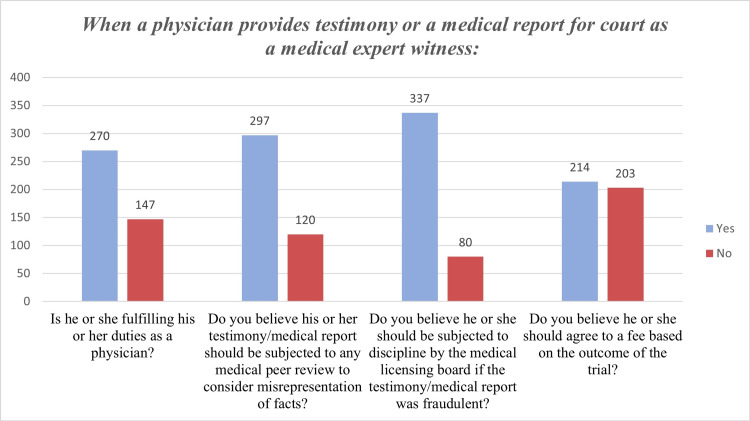
Perception of physicians towards medicolegal scenarios.

Regarding the perception of physicians towards various medicolegal statements, the statement that physicians should be subjected to discipline by the medical licensing board if the testimony/medical report was fraudulent received the highest rate of agreement (80%). Participants were nearly divided on whether physicians should accept compensation depending on the trial's outcome. Around 35% of participants felt that providing court testimony or a medical report is not part of a physician's responsibilities. Nonetheless, 71% of respondents agreed that testimony/medical reports should be subjected to peer review to avoid misrepresenting facts. Figure 2 illustrates the perceptions of physicians toward medicolegal scenarios.

## Discussion

This article describes physicians' experiences, education, training, willingness, self-competence, attitude, and perception as expert witnesses in Saudi Arabia. To our understanding, this is the first study in Saudi Arabia exploring physicians' role as expert witnesses. Our findings indicate that physicians' involvement in the legal system, whether through providing medical reports or testimony, is limited. While physicians are interested in learning and providing legal services in their respective fields, they lack sufficient education and experience to develop and provide court reports and testimonies.

The state of the medical expert witness

The increasing number of malpractice lawsuits in Saudi Arabia, with a 26% increase from 2016 to 2018, highlights the importance of involving physicians' expertise in the legal system [13]. Currently, the utilization of medico-legal expertise in courtrooms is mainly limited to forensic medicine specialists [14]. The Code of Ethics for Healthcare Practitioners emphasizes the responsibility of healthcare practitioners in judiciary proceedings. However, our findings suggest a discrepancy between this ideal and the reality of physicians' legal involvement. The Medico-Legal Committee (MLC) receives and investigates professional malpractice claims resulting in patient harm or death. The MLC conducts a comprehensive review of documents, medical files, and interviews with the plaintiff and defendant to reach a final decision [15]. However, issues arise when the MLC members may not have the same medical specialty as the case under review, making it challenging to establish an objective opinion based on the latest knowledge and practice. This is particularly relevant in high-risk specialties such as OB-GYN, which require specialized expertise to provide objective evidence-based opinions [13]. Therefore, we recommend providing all physicians from various specialties with the opportunity to participate in legal cases, along with education and training, to enhance their effectiveness in the legal field.

Guidelines and certification procedures

To our knowledge, there are no established guidelines or licensing procedures for physicians in Saudi Arabia to become expert witnesses. Globally, some medical societies have provided guidelines and certification procedures for expert witnesses, addressing potential issues and ensuring appropriate training [14]. However, these guidelines may lack certain elements, such as specifying principles for appropriate compensation. In our study, participants had differing opinions on whether physicians should accept compensation based on the trial's outcome. Therefore, it is recommended to establish local guidelines and certification procedures in Saudi Arabia that address and improve upon any deficiencies in international guidelines. This would enable physicians to become certified medical expert witnesses in a standardized manner.

Recommendations

Based on our evaluation and the existing literature, we propose the following recommendations to enhance the role of medical expert witnesses in Saudi Arabia. Firstly, there is a need to develop evidence-based guidelines tailored for physicians serving as expert witnesses. Secondly, standardizing the qualifications and certifications required for medical expert witnesses across the country is essential. Thirdly, fostering collaboration and communication between medical expert witnesses and legal professionals is crucial to ensure a comprehensive understanding of the legal system and the role of expert witnesses. Additionally, there should be ongoing training and professional development opportunities provided to medical expert witnesses to keep them abreast of best practices and advancements in the field. Moreover, introducing medicolegal education for physicians through courses, lectures, and workshops would be beneficial. Lastly, it is imperative to incorporate medical law curricula into both medical and law schools nationwide to equip future professionals with the necessary knowledge and skills in this domain.

Limitations

There are various limitations to this paper that should be highlighted. Because the participants provided the information, there is a risk of self-report and recall bias. The role of physicians as medical expert witnesses in legal proceedings is somewhat contentious, particularly in medical malpractice cases in which physicians express their opinions on the quality of care provided by their colleagues [16]. Furthermore, because this is the first study in the country that has addressed this subject, comparison with other papers was not possible.

## Conclusions

Overall, a large percentage of physicians have never possessed prior education or training as expert witnesses, nor have they participated as one in any legal proceedings. The majority of physicians were interested in learning or training as an expert witness and were willing to provide a medical report or testimony for court. The findings highlight the need for increased engagement and experience among physicians in serving as expert witnesses and the potential gaps in education and training. Tailoring programs to different professional levels and providing early and mid-career physicians with targeted support may enhance physicians' confidence and competence as expert witnesses. Furthermore, the study's findings underscore the importance of continued efforts to ensure ethical and accountable practices in the medicolegal domain. Further research and interventions are warranted to enhance physicians' engagement, competence, and ethical practices as expert witnesses in Saudi Arabia.
